# Impact of the coronavirus disease 2019 (COVID-19) pandemic on the diagnosis of neck and low back pain in outpatient practices in Germany

**DOI:** 10.1016/j.pmedr.2022.102096

**Published:** 2022-12-19

**Authors:** Louis Jacob, Hans Oh, Lee Smith, Ai Koyanagi, Marcel Konrad, Karel Kostev

**Affiliations:** a Research and Development Unit, Parc Sanitari Sant Joan de Déu, CIBERSAM, Dr. Antoni Pujadas, 42, Sant Boi de Llobregat, Barcelona, Spain; b Department of Physical and Rehabilitation Medicine, Lariboisière-Fernand Widal Hospital, AP-HP, University Paris Cité, Paris, France; c Faculty of Medicine, University of Versailles Saint-Quentin-en-Yvelines, Montigny-le-Bretonneux, France; d Suzanne Dworak Peck School of Social Work, University of Southern California, Los Angeles, CA, USA; e Centre for Health, Performance, and Wellbeing, Anglia Ruskin University, Cambridge, United Kingdom; f Institució Catalana de Recerca i Estudis Avançats (ICREA), Pg. Lluis Companys 23, Barcelona, Spain; g FOM University of Applied Sciences for Economics and Management, Frankfurt, Germany; h Epidemiology, IQVIA, Frankfurt, Germany

**Keywords:** COVID-19, Neck pain, Low back pain, General practices, Orthopedic practices, Germany

## Abstract

Little is known about how COVID-19 has impacted the diagnosis of neck and low back pain in outpatient practices in Germany. Thus, this study aimed to compare the number of new diagnoses of neck and low back pain in German general and orthopedic practices between 2020 and 2021 and 2019. This retrospective study included patients aged ≥ 18 years with at least one visit to one of 915 general and 145 orthopedic practices in Germany in March – December 2019 (N = 2,842,145), March – December 2020 (N = 2,810,179), or March – December 2021 (N = 3,214,419). The number of patients newly diagnosed with neck and low back pain per general and orthopedic practice was compared between March – December 2020 and March – December 2019, and between March – December 2021 and March – December 2019 using Wilcoxon signed-rank tests. Analyses were conducted in general and orthopedic practices separately and were also stratified by sex and age. There was a decrease in new diagnoses of neck and low back pain in general and orthopedic practices between 2020 and 2019, and between 2021 and 2019. This decrease reached statistical significance for neck pain in general practices in 2020 (−12.4 %) and 2021 (−6.1 %), and for low back pain in general practices in 2020 (−9.3 %). Similar findings were obtained in sex- and age-stratified analyses. The COVID-19 pandemic had a negative impact on the diagnosis of neck and low back pain in general and orthopedic practices in Germany. More data from other settings and countries are warranted to confirm or refute these results.

## Highlights

• This study was conducted in general and orthopedic practices in Germany in 2019–2021.
• New diagnoses of neck pain decreased in Germany in 2020 and 2021 compared with 2019.
• There was also a decrease in the number of new diagnoses of low back pain.
• These findings were corroborated in the sex- and age-stratified analyses.

## 1 Introduction

Coronavirus disease 2019 (COVID-19) is a global pandemic caused by the severe acute respiratory syndrome coronavirus 2 (SARS-CoV-2) (da Rosa Mesquita et al., 2020). As of December 2, 2022, 640 million people globally have contracted COVID-19, while approximately 6.6 million people have died from the disease (World Health Organization, 2022). In Germany, the number of COVID-19 cases and related deaths is around 36.5 million and 158,000, respectively. To decrease the risk of SARS-CoV-2 transmission, public health measures have been undertaken, and these measures have included, for example, mask wearing, physical distancing, and lockdowns (Talic et al., 2021).

Despite this international effort, healthcare systems have been rapidly overwhelmed (Tangcharoensathien et al., 2021), and this disruption has had deleterious effects on the diagnosis of several life-threatening disorders (e.g., stroke (Tejada Meza et al., 2020) and myocardial infarction (Watanabe et al., 2021)). The decreased diagnosis of these diseases since the beginning of the pandemic may further be explained by the avoidance of healthcare systems because of fear of contracting SARS-CoV-2 (Czeisler et al., 2020). Interestingly, COVID-19 has also negatively impacted the diagnosis of other conditions such as musculoskeletal diseases. For example, one study, using data obtained at two tertiary care centers in the United States, found a decreasing trend in the number of new cases of multiple musculoskeletal conditions (e.g., knee osteoarthritis, shoulder sprain, and carpal tunnel syndrome) (Yu et al., 2021). Less is known about how the COVID-19 crisis has affected the diagnosis of neck and low back pain, although some literature indicates that the pandemic has led to an increase in the prevalence of self-reported low back pain (Papalia et al., 2022) and an aggravation of symptoms in people already affected by low back pain (Bailly et al., 2022). To the best of the authors’ knowledge, the three only studies on this topic have been conducted in hospital settings (Borsa et al., 2020, Jäntti et al., 2022, Sharma et al., 2021), and no data is available from primary care and specialized practices. This lack of data is of concern because the prevalence of self-reported musculoskeletal pain has been high in the worldwide population since the beginning of the COVID-19 pandemic (Papalia et al., 2022, Šagát et al., 2020), while neck and low back pain are risk factors for physical disability (Wu et al., 2020), poor mental health (Demyttenaere et al., 2007), and low quality of life (Pedisic et al., 2013). The high prevalence mentioned above may be explained by multiple factors such as low physical activity (Alzahrani et al., 2019, Roggio et al., 2021, Sonza et al., 2021, Wilke et al., 2021), increased stress (Choi et al., 2021, Xiong et al., 2020) and increased smoking (Bar-Zeev et al., 2022, Shiri et al., 2010).

Therefore, this study aimed to compare the number of new diagnoses of neck and low back pain in general and orthopedic practices in Germany between 2020 and 2021 (i.e., the COVID-19 era) and 2019 (i.e., the pre-COVID-19 era). It was hypothesized that the number of patients newly diagnosed with neck and low back pain significantly decreased in 2020 and 2021 compared with 2019. If the hypothesis of the study is confirmed, this decreasing trend would suggest that a substantial proportion of neck and low back pain cases would have been undiagnosed, potentially undermining the management and treatment of these musculoskeletal conditions in the German general population.

## 2 Methods

### 2.1 Database

This study used data from the Disease Analyzer database (IQVIA). This database has been described in detail elsewhere (Rathmann et al., 2018). The Disease Analyzer database contains demographic, diagnosis, and prescription data anonymously obtained from general and specialized practices. Diagnosis data are coded using the International Classification of Diseases, 10th revision (ICD-10), while prescription data are coded using the Anatomical Classification of Pharmaceutical Products of the European Pharmaceutical Market Research Association (EphMRA). The quality of the data is assessed on a regular basis using several criteria, such as completeness of documentation and linkage between diagnoses and prescriptions. Practices included in the Disease Analyzer database are selected based on physician age, specialty group, community size category, and German federal state. Finally, around 3 % of practices in Germany are included in the Disease Analyzer database, and this database has been found to be representative of all practices in the country (Rathmann et al., 2018).

### 2.2 Study population

This retrospective study included all patients aged ≥ 18 years with at least one visit to one of 915 general and 145 orthopedic practices in Germany in March – December 2019, March – December 2020, or March – December 2021. Only practices continuously delivering data between April 2018 and December 2021 were included. Patients had to have no diagnosis of neck (ICD-10 code: M54.2) and low back pain (ICD-10 code: M54.5) prior to the study period of interest (i.e., March – December 2019, March – December 2020, or March – December 2021).

### 2.3 Diagnosis of neck and low back pain

The diagnosis of neck and low back pain was exclusively based on ICD-10 codes. For neck pain, the ICD-10 code was M54.2, including all types of cervicalgia except those due to intervertebral cervical disc disorder. For back pain, the ICD-10 code was M54.5, including all types of low back pain except lumbago with sciatica and lumbago due to intervertebral disc displacement.

### 2.4 Statistical analyses

The number of patients newly diagnosed with neck and low back pain per general and orthopedic practice was compared between March – December 2020 and March – December 2019, and between March – December 2021 and March – December 2019 using Wilcoxon signed-rank tests. Analyses were conducted in general and orthopedic practices separately and were also stratified by sex and age. P-values < 0.05 were considered statistically significant. Analyses were performed using SAS 9.4.

### 2.5 Statement of ethics

German law allows the use of anonymous electronic medical records for research purposes under certain conditions. According to this legislation, it is not necessary to obtain informed consent from patients or approval from a medical ethics committee for this type of observational study that contains no directly identifiable data.

## 3 Results

There were 2,161,520 patients followed in general practices and 680,625 patients followed in orthopedic practices in March – December 2019. Respective numbers were 2,169,874 and 640,305 for March – December 2020, and 2,550,751 and 663,668 for March – December 2021. The overall difference in the number of patients newly diagnosed with neck and low back pain per practice is displayed in Table 1. There was a decrease in new diagnoses of neck and low back pain in general and orthopedic practices between 2019 and 2020, and between 2019 and 2021. This decrease reached statistical significance for neck pain in general practices in 2020 (−12.4 %) and 2021 (−6.1 %), and for low back pain in general practices in 2020 (−9.3 %). Sex- and age-stratified analyses are shown in Tables 2 and 3, respectively. The number of men and women newly diagnosed with neck and low back pain per practice decreased in 2020 and 2021 compared with 2019. In men, differences were only significant for neck pain in general practices in 2020 versus 2019 (−10.3 %). In women, differences were significant for neck pain in general practices in 2020 versus 2019 (−13.7 %), neck pain in general practices in 2021 versus 2019 (−9.8 %), and low back pain in general practices in 2020 versus 2019 (−10.8 %). In terms of age, the number of patients newly diagnosed with neck and low back pain per practice decreased in most age groups, and this decrease was particularly high and reached significance for neck pain in patients aged 61–70 years in general practices in 2020 versus 2019 (−13.6 %), neck pain in patients aged greater than 70 years in general practices in 2020 versus 2019 (−13.5 %), and neck pain in patients aged 41–50 years in general practices in 2020 versus 2019 (−12.7 %).

**Table 1:** Overall difference in the number of patients newly diagnosed with neck and low back pain per practice in Germany between 2020 and 2021 and 2019.

Practice	Disorder	Mar – Dec 2019	Mar – Dec 2020	Mar – Dec 2021	2020 versus 2019 (%)	P-value	2021 versus 2019 (%)	P-value
General	Neck pain	36.9 (39.4)	32.3 (38.6)	34.6 (55.1)	**−12.4**	**0.003**	**−6.1**	**0.039**
	Low back pain	45.0 (44.9)	40.8 (40.2)	43.3 (46.5)	**−9.3**	**0.045**	−3.7	0.161

Orthopedic	Neck pain	170.4 (155.0)	160.2 (165.6)	157.6 (153.7)	−6.0	0.417	−7.5	0.398
	Low back pain	206.2 (200.8)	192.6 (200.5)	200.6 (219.3)	−6.6	0.493	−2.7	0.521

Data are mean (standard deviation).

P-values were obtained using Wilcoxon signed-rank tests.

Significant differences are in bold.

**Table 2:** Sex-stratified differences in the number of patients newly diagnosed with neck and low back pain per practice in Germany between 2020 and 2021 and 2019.

Sex	Practice	Disorder	Mar – Dec 2019	Mar – Dec 2020	Mar – Dec 2021	2020 versus 2019 (%)	P-value	2021 versus 2019 (%)	P-value
Male	General	Neck pain	14.9 (17.1)	13.3 (17.8)	14.8 (33.0)	**−10.3**	**0.010**	−0.7	0.138
		Low back pain	23.8 (24.6)	21.9 (22.3)	23.3 (26.1)	−8.0	0.088	−2.0	0.228
	Orthopedic	Neck pain	61.4 (57.2)	59.6 (63.4)	58.8 (59.3)	−2.9	0.526	−4.1	0.491
		Low back pain	90.5 (87.3)	85.7 (87.8)	88.3 (96.7)	−5.3	0.577	−2.5	0.473

Female	General	Neck pain	22.0 (23.2)	19.0 (21.6)	19.9 (24.3)	**−13.7**	**0.001**	**−9.8**	**0.014**
		Low back pain	21.2 (21.5)	18.9 (18.9)	20.0 (21.4)	**−10.8**	**0.036**	−5.6	0.146
		Neck pain	109.1 (99.3)	100.7 (103.5)	98.7 (96.0)	−7.7	0.345	−9.5	0.330
	Orthopedic	Low back pain	115.7 (115.4)	106.9 (114.1)	112.4 (124.6)	−7.6	0.460	−2.9	0.548
						

Data are mean (standard deviation).

P-values were obtained using Wilcoxon signed-rank tests.

Significant differences are in bold.

**Table 3:** Age-stratified differences in the number of patients newly diagnosed with neck and low back pain per practice in Germany between 2020 and 2021 and 2019.

Age	Practice	Disorder	Mar – Dec 2019	Mar – Dec 2020	Mar – Dec 2021	2020 versus 2019 (%)	P-value	2021 versus 2019 (%)	P-value
18–30 years	General	Neck pain	6.6 (9.5)	5.8 (9.9)	6.4 (13.9)	**−11.1**	**0.025**	−2.4	0.105
		Low back pain	7.9 (11.0)	6.9 (9.2)	7.7 (10.9)	**−12.6**	**0.041**	−1.9	0.378
	Orthopedic	Neck pain	20.8 (21.4)	21.7 (26.3)	19.8 (22.3)	4.4	0.837	−4.8	0.467
		Low back pain	26.7 (28.0)	24.4 (27.3)	26.2 (31.0)	−8.4	0.672	−1.9	0.569

31–40 years	General	Neck pain	6.8 (8.9)	6.0 (8.3)	6.5 (12.4)	**−11.8**	**0.047**	−3.3	0.304
		Low back pain	7.6 (9.2)	7.2 (8.6)	7.8 (10.0)	−5.4	0.205	1.8	0.716
	Orthopedic	Neck pain	26.5 (26.2)	26.5 (29.8)	26.5 (29.2)	0.2	0.834	0.1	0.693
		Low back pain	31.1 (31.1)	29.1 (31.6)	30.4 (35.2)	−6.5	0.646	−2.2	0.604

41–50 years	General	Neck pain	6.6 (8.3)	5.8 (8.3)	6.1 (11.2)	**−12.7**	**0.005**	**−7.1**	**0.029**
		Low back pain	7.6 (8.3)	7.0 (8.0)	7.5 (8.9)	−8.2	0.062	−1.8	0.461
	Orthopedic	Neck pain	31.2 (29.0)	30.2 (31.6)	29.3 (29.6)	−3.2	0.593	−6.1	0.425
		Low back pain	34.7 (33.9)	31.5 (32.7)	33.6 (37.5)	−9.3	0.410	−3.1	0.477

51–60 years	General	Neck pain	7.4 (7.8)	6.5 (7.3)	6.8 (9.7)	**−12.2**	**0.008**	−7.8	0.052
		Low back pain	9.4 (9.1)	8.5 (8.4)	8.9 (9.7)	−9.1	0.075	−4.7	0.127
	Orthopedic	Neck pain	40.1 (37.3)	38.9 (40.7)	37.2 (36.3)	−3.0	0.504	−7.2	0.459
		Low back pain	45.6 (46.0)	44.5 (47.7)	44.8 (50.1)	−2.5	0.666	−1.8	0.493

61–70 years	General	Neck pain	4.5 (4.7)	3.9 (4.4)	4.3 (6.4)	**−13.6**	**0.007**	−5.4	0.134
		Low back pain	5.7 (6.9)	5.2 (5.6)	5.5 (6.2)	−9.2	0.101	−4.5	0.256
	Orthopedic	Neck pain	25.5 (24.9)	22.2 (23.1)	23.3 (23.6)	−13.0	0.243	−8.8	0.428
		Low back pain	31.0 (32.9)	30.0 (33.2)	31.4 (36.1)	−3.4	0.660	1.1	0.730

greater than70 years	General	Neck pain	5.0 (5.1)	4.3 (4.6)	4.4 (5.1)	**−13.5**	**0.003**	**−11.7**	**0.010**
		Low back pain	6.8 (8.6)	6.0 (7.0)	6.0 (8.1)	**−11.5**	**0.028**	**−12.2**	**0.006**
	Orthopedic	Neck pain	26.4 (29.4)	20.8 (23.7)	21.6 (23.7)	−21.5	0.102	−18.3	0.170
		Low back pain	37.1 (43.6)	33.2 (39.6)	34.3 (42.3)	−10.7	0.358	−7.7	0.418

Data are mean (standard deviation).

P-values were obtained using Wilcoxon signed-rank tests.

Significant differences are in bold.

## 4 Discussion

### 4.1 Main findings

This retrospective study, including 915 general and 145 orthopedic practices from Germany, revealed that the number of patients newly diagnosed with neck and low back pain per practice decreased in March – December 2020 and March – December 2021 compared with March – December 2019. This decrease reached statistical significance for both neck and low back pain in general practices in March – December 2020 versus March – December 2019. These findings were corroborated in the sex- and age-stratified analyses. To the best of the authors’ knowledge, this is the first study to have investigated the impact of the COVID-19 pandemic on the diagnosis of neck and low back pain in primary care and specialized practices.

### 4.2 Interpretation of findings

The present results are in line with previous studies conducted in hospital settings. For example, it was observed in four hospitals in Italy that the number of visits for acute low back pain to emergency and outpatient departments decreased by 87.2 % in March – April 2020 compared with the same period in 2019 (Borsa et al., 2020). Another retrospective study conducted in three public hospitals in Australia found a 31 % decrease in the number of presentations for spinal disorders in March – May 2020 versus March – May 2019 (Sharma et al., 2021). A third study, using data collected in three large hospitals in Finland, revealed that the number of emergency department visits per month for back pain significantly decreased during the lockdown period and when regional restrictions were applied in 2020 compared with the corresponding months in 2017–2019 (Jäntti et al., 2022). The present study from Germany adds to the literature by showing that the COVID-19 crisis has also negatively impacted the diagnosis of neck and low back pain in private practices and that a substantial proportion of people with these musculoskeletal conditions were likely left undiagnosed. Besides, these data suggest that the deleterious effects of COVID-19 on the diagnosis of neck and low back pain in these practices have been particularly strong during the first year of the pandemic (i.e., 2020) and were less pronounced during the second year (i.e., 2021). The fact that the decrease in the number of patients newly diagnosed with neck and low back pain per practice was significant in general but not in orthopedic practices may be related to the sample size of this study. There were 915 general and 145 orthopedic practices available for analysis, and analyses of data obtained in orthopedic practices may have lacked statistical power.

There are at least two hypotheses to explain the results of this study. First, a substantial body of research has shown that healthcare systems have been overwhelmed in the first months of the COVID-19 pandemic. One study of more than 500 general practices from Ireland found that the median number of face-to-face consultations decreased from 26 in February 2020 to 8 in June 2020 (Homeniuk and Collins, 2021). Another survey carried out in the United States in 2020, including 99 orthopedic surgeons, revealed that more than 90 % of them reported decreased patient volume after the COVID-19 outbreak (Kale et al., 2020). In this context, general practitioners and orthopedic surgeons may have prioritized urgent consultations and postponed other consultations. Interestingly, data obtained at an orthopedic center in Italy identified major changes in clinical activities since the beginning of the COVID-19 crisis, with an increase in emergency admissions from 158 in 2019 to 268 in 2020 and a decrease in planned surgical admissions from 2,172 to 664 (Zagra et al., 2020). The second hypothesis is that patients may have feared contracting COVID-19 at the hospital and in private practices, and may have delayed non-urgent and also urgent consultations. In 4,975 adults living in the United States, the prevalence of avoidance of medical care during the pandemic because of concerns about COVID-19 was 40.9 %, and this figure was 12.0 % for urgent emergency care and 31.5 % for routine care (Czeisler et al., 2020). These findings were corroborated in a population-based cross-sectional study conducted in the Netherlands (N = 5,656), as healthcare avoidance was reported by 20.2 % of participants and was higher in those with fear of contracting COVID-19 (Splinter et al., 2021).

### 4.3 Public health implications and directions for future research

Based on this study, the number of new diagnoses of neck and low back pain decreased in general and orthopedic practices in Germany in 2020 and 2021 compared with 2019. This decrease is of particular concern, because other bodies of research have found high rates of self-reported musculoskeletal pain in the general population since the beginning of the pandemic (Papalia et al., 2022, Šagát et al., 2020). Furthermore, post-COVID-19 syndrome following SARS-CoV-2 infection can also include musculoskeletal pain (Aiyegbusi et al., 2021, Karaarslan et al., 2022). In this context, lack of diagnosis or delayed diagnosis may have been common, and patients with neck and low back pain may have frequently been denied medical reassurance, education, and adequate treatment (e.g., oral analgesics, topical medications, and exercise therapy) (Corp et al., 2021). Exercise is a key player in the management of neck and low back pain, and regular exercise has been found to reduce disability in people with low back pain in Germany during the COVID-19 crisis (Hochheim et al., 2022). Given that there is uncertainty on how COVID-19 will impact healthcare systems in the future, although efforts have been made to predict the dynamics of the COVID-19 pandemic (Davahli et al., 2021), measures should be taken to mitigate the deleterious effects of this health crisis on the diagnosis of musculoskeletal conditions. One key measure is the development and implementation of teleconsultations, which may allow the early diagnosis and management of neck and low back pain in populations with limited access to face-to-face consultations (Yedlinsky and Peebles, 2021). Finally, in terms of future research, more data from other countries and settings are warranted to confirm or refute these results. Moreover, future studies should investigate the respective and relative role played by the overwhelming of healthcare systems and fear of COVID-19 in the decreased number of diagnoses of neck and low back pain observed in this research.

### 4.4 Strengths and limitations

The strengths of this study are the number of patients and practices included in the analyses, as well as the use of data collected until December 2021. Nonetheless, the study results should be interpreted in light of its limitations. First, patients may have been diagnosed with neck and low back pain in other settings (e.g., at the hospital or in rheumatology practices), and the findings may thus not be extrapolated to the German population. Second, the diagnosis of neck and low back pain relied exclusively on ICD-10 codes, and data on the severity of pain and the duration of symptoms were unavailable. Third, no information is available in the Disease Analyzer database on education level, employment, and income, although it is possible that the impact of COVID-19 on the diagnosis of neck and low back pain is different between patients with high and those with low socioeconomic status.

## 5 Conclusions

This study, using data from general and orthopedic practices in Germany, found that the number of new diagnoses of neck and low back pain has decreased since the beginning of the COVID-19 pandemic. The present findings suggest that a considerable proportion of people with neck and low back pain may have been undiagnosed, and this decreasing trend could have long-lasting deleterious effects on the management of these musculoskeletal disorders in this country. More data are warranted to confirm or refute these results in other countries.

Statements and declarations

## Disclosure of funding

This research did not receive any specific grant from funding agencies in the public, commercial, or not-for-profit sectors.

## Declaration of Competing Interest

The authors declare that they have no known competing financial interests or personal relationships that could have appeared to influence the work reported in this paper.

## Data Availability

Data will be made available on request.
